# Exercise intensity prescription in cardiovascular rehabilitation: bridging the gap between best evidence and clinical practice

**DOI:** 10.3389/fcvm.2024.1380639

**Published:** 2024-08-27

**Authors:** Juliana Goulart Prata Oliveira Milani, Mauricio Milani, Kenneth Verboven, Gerson Cipriano, Dominique Hansen

**Affiliations:** ^1^ Rehabilitation Research Center (REVAL), Faculty of Rehabilitation Sciences, Hasselt University, Hasselt, Belgium; ^2^ Graduate Programme in Health Sciences and Technologies, University of Brasilia (UnB), Brasilia, Brazil; ^3^ Heart Centre Hasselt, Jessa Hospital, Hasselt, Belgium; ^4^ Faculty of Medicine and Life Sciences, Biomedical Research Institute (BIOMED), Hasselt, Belgium; ^5^ Graduate Program in Human Movement and Rehabilitation of Evangelical (PPGMHR), UniEVANGÉLICA, Anápolis, Brazil

**Keywords:** exercise, physical activity, cardiac rehabilitation, cardiovascular health, cardiovascular disease, heart disease risk factors, exercise test, health

## Abstract

Optimizing endurance exercise intensity prescription is crucial to maximize the clinical benefits and minimize complications for individuals at risk for or with cardiovascular disease (CVD). However, standardization remains incomplete due to variations in clinical guidelines. This review provides a practical and updated guide for health professionals on how to prescribe endurance exercise intensity for cardiovascular rehabilitation (CR) populations, addressing international guidelines, practical applicability across diverse clinical settings and resource availabilities. In the context of CR, cardiopulmonary exercise test (CPET) is considered the gold standard assessment, and prescription based on ventilatory thresholds (VTs) is the preferable methodology. In settings where this approach isn't accessible, which is frequently the case in low-resource environments, approximating VTs involves combining objective assessments—ideally, exercise tests without gas exchange analyses, but at least alternative functional tests like the 6-minute walk test—with subjective methods for adjusting prescriptions, such as Borg's ratings of perceived exertion and the Talk Test. Therefore, enhancing exercise intensity prescription and offering personalized physical activity guidance to patients at risk for or with CVD rely on aligning workouts with individual physiological changes. A tailored prescription promotes a consistent and impactful exercise routine for enhancing health outcomes, considering patient preferences and motivations. Consequently, the selection and implementation of the best possible approach should consider available resources, with an ongoing emphasis on strategies to improve the delivery quality of exercise training in the context of FITT-VP prescription model (frequency, intensity, time, type, volume, and progression).

## 1 Introduction

Consistently engaging in physical activity (PA) stands out as the most effective approach to preventing and managing non-communicable diseases, including cardiovascular diseases (CVD), while ensuring overall health (1). A variety of documented health benefits, such as improved quality of life and reduced cardiovascular and overall mortality, are achieved through regular PA and structured exercise in cardiovascular rehabilitation (CR) (2–4). Therefore, exercise training is strongly advised in primary and secondary prevention guidelines for CVD and is considered an essential component of healthcare plans for individuals with this profile (5–7).

The current recommendations from the World Health Organization (WHO) emphasize the importance of intensity as a significant component of exercise prescription for achieving meaningful health benefits. These recommendations state that adults should engage in a minimum of 150–300 min of moderate-intensity endurance physical activity per week, or at least 75–150 min of vigorous-intensity endurance physical activity per week, or an equivalent combination (8). Even greater volumes of PA are advised when greater health benefits are ambitioned (e.g., fat mass loss) (1, 9). Additionally, in the context of CR programs, although structured endurance exercise typically ranges from 24 to 30 min, extending these sessions to 35–45 min has been suggested to yield better outcomes across various cardiovascular populations (10–12).

For a patient at risk for (e.g., a person with obesity or diabetes) or with CVD, a tailored intensity prescription aims to optimize the benefits of the exercise intervention while minimizing associated risks (6, 13–15). Prescribing exercise training should align with the FITT-VP model, encompassing factors such as frequency, intensity, duration (time), specific type of exercise, volume, and progression (16, 17). Within this framework, establishing the ideal endurance exercise intensity is a cornerstone in enhancing intervention results (7, 18–21). However, it has become clear that clinicians and healthcare professionals still struggle with this task (7).

Therefore, the present document aims to offer a practical and critically revised update on assessing and determining the most optimal endurance exercise intensity in various settings for health professionals engaged in CR. Additionally, the document will offer a worldwide overview of the current exercise intensity domains recommended by CR guidelines, along with the application of different methods in clinical cases. Hence, it aims to bridge the gaps between best evidence and clinical practice, considering different available resources in the context of endurance exercise prescription for CR.

## 2 The importance of endurance exercise intensity in cardiovascular rehabilitation

Evidence indicates that one metabolic equivalent of tasks (MET) increase in exercise capacity during CR is linked to a 13% reduction in mortality (22). Recently, Kokkinos et al. (23), in a study involving 750,302 adults, reported an inverse and graded association between cardiorespiratory fitness (CRF), determined by peak MET (MET_peak_), and mortality risk across the age spectrum, among men, women, and all races independently. In this study, individuals with the lowest CRF exhibited a 4-fold higher mortality risk than those with the highest fitness levels. Even in obese individuals, it has been shown that increments in MET_peak_ during follow-up relate to reductions in mortality, but weight loss did not show such an association (24). Hence, in terms of prognosis “fitness” seems more important than “fatness”.

Optimizing exercise prescription is therefore becoming a growing goal, with an increasing interest in identifying factors contributing to changes in CRF throughout CR programs (25). Iannetta et al. (26) examined 2,310 CVD patients who completed a 12-week center-based CR program, and reported that the applied exercise intensity was one of the main predictors of MET_peak_ post-CR, along with baseline MET_peak_, training duration, body mass index, and age. Uddin et al. (4), in a meta-regression analysis of 51 trials, including 7,553 patients with coronary artery disease (CAD) and heart failure (HF), reported that for each 10% increase in the applied exercise intensity, measured as percentual of peak oxygen uptake (%VO_2peak_) or percentual of peak heart rate (%HR_peak_), a more substantial average improvement in peak oxygen uptake (VO_2peak_) by 1.0 ml/kg/min was noted across trials. This means that a large increment in exercise intensity must be exert (e.g., by 20%) to induce significantly greater improvements in VO_2peak_ (e.g., +2 ml/kg/min). Another meta-analysis involving 13,220 patients from 128 studies found that prescribing higher exercise intensities during CR resulted in the most significant pooled effect for the change in relative VO_2peak_. Nevertheless, while all effects were statistically significant, they exhibited high heterogeneity, and the comparisons of intensities may lack clinical significance, with moderate, moderate-to-vigorous, and vigorous interventions showing a mean increase, expressed as ±95% CI, of 4.1 ± 2.3, 4.9 ± 0.9, and 5.5 ± 1.3 ml/kg/min, respectively (27).

In addition, exercise intensity matters for fat mass loss and weight control. A recent meta-analysis (involving 2,190 participants from 40 randomised controlled trials) demonstrated a dose-response effect of −0.15 (−0.23 to −0.07; *p* < 0.001) per 1,000 calories deficit per week for visceral fat mass loss in overweight/obese persons (28). Regarding lipid profile, in a systematic review with meta-analysis and meta-regression involving 2,990 sedentary adults with three or more metabolic syndrome factors, endurance exercise training significantly improved all lipid parameters (*P* < 0.001), with intensity associated with changes in triglycerides and volume associated with high-density lipoprotein-cholesterol (HDL-C) and low-density lipoprotein-cholesterol (LDL-C) (29). Another meta-analysis of randomized controlled trials showed that endurance exercise (at least moderate intensity, ≥12 weeks) positively affects lipid biomarkers linked to cardiovascular disease risk in sedentary adults by reducing atherogenic apolipoproteins, lipoprotein sub-fractions, and atherogenic lipid ratios while increasing antiatherogenic apolipoproteins and lipoprotein subfractions (30).

For significant blood pressure reductions, a sufficiently endurance exercise intensity (at moderate to high levels) must also be applied (31), and comparisons between different exercise types have been studied. A recent systematic review and meta-analysis found that both high-intensity interval training (HIIT) and moderate-intensity continuous training (MICT) reduce systolic blood pressure in hypertensive individuals, with HIIT showing greater reductions in diastolic blood pressure and potentially greater improvements in VO_2peak_ compared to MICT (32). Other meta-analyses reported comparable reductions in blood pressure in adults with pre- to established hypertension for both modalities, with HIIT again showing greater improvements in VO_2peak_ compared to MICT (33). Similar findings emerged in a separate meta-analysis of 10 studies involving 266 older adults where both HIIT and MICT demonstrated comparable reductions in blood pressure (34). The exercise-related reductions in blood pressure have not been fully elucidated and involve a combination of various mechanisms that differ among individuals, populations, and exercise training protocols (35, 36). A recent meta-analysis of 18 trials, involving 803 participants undergoing various exercise regimens (e.g., endurance, resistance, or combined) for at least 4 weeks, revealed significant reductions in systolic and diastolic blood pressure (mean difference: −6.2 mmHg and −4.5 mmHg, respectively) (37). These reductions were accompanied by mechanisms involving positive modulation of the renin–angiotensin–aldosterone system and sympathetic nervous system activity, resulting in decreased plasma angiotensin-II, aldosterone, and norepinephrine levels. Other mechanisms may include, for instance, local vascular resistance regulation influenced by endothelium-driven factors (36, 38), with an increase in nitric oxide (a potent vasodilator) (38, 39) and a reduction in endothelin-1 (a potent vasoconstrictor) (39, 40).

Significantly, HIIT protocols can vary widely, and exercise approaches exhibit inadequate reporting across diverse health conditions. Such unclear prescription and delivery methods affect the understanding of the “dose” of exercise needed to optimize health outcomes and, consequently, may limit its translation from research to practical application.

In a meta-analysis of 24 studies (involving 1,080 participants), HIIT demonstrated significantly greater improvement in VO_2peak_ compared to MICT (+1.40 ml/min/kg for HIIT; 95% CI: 0.69–2.11; *P* < 0.001). This improvement persisted when examining patients with CAD and HF with reduced ejection fraction (HFrEF) separately, but was evident only with higher total energy expenditure (41). In another meta-analysis of 664 CAD and HF patients (15 studies), HIIT outperformed MICT in improving VO_2peak_ for CAD, with no significant difference in HF. The most significant VO_2peak_ improvement with HIIT occurred within eight weeks, and this modality also led to significantly greater increases in the first ventilatory threshold (VT_1_) and in left ventricular ejection fraction (LVEF) compared to MICT, with no significant differences in the ventilatory efficiency (VE/VCO_2_ slope). Yue et al. (42), in a meta-analysis of twenty-two studies (949 participants), concluded that HIIT is safe and more effective than MICT for improving CRF in CVD patients. Notably, HIIT performed three times weekly for over 12 weeks resulted in the greatest improvements.

Conversely, other meta-analyses, including CAD patients (12 studies, *n* = 609 patients) (43), and HFrEF patients (13 studies, *n* = 411 patients) (44), found that HIIT was not superior to MICT in increasing VO_2peak_ when isocaloric exercise training programs were compared. Hence, it seems that HIIT could offer a more time-efficient method for improving VO_2peak_, considering that the total exercise duration was considerably shorter compared to MICT (45, 46). So, it is reasonable that all CR programs should consider the incorporation of HIIT as a supplementary or alternative for traditional MICT (44, 47, 48), not solely because of the described time-efficiency, but also due to its potential for superior or at least equivalent benefits compared to MICT in selected patients, as well as its positive impact on adherence for those who find it more motivating.

Moderate-intensity interval training (MIIT) has been proposed as a feasible and effective alternative, as reported, for instance, in community-based cardiac maintenance programs for patients with CVD, improving exercise performance and regulating blood glucose levels (49). Additionally, implementing MIIT before HIIT in training programs can be a valid strategy to boost body adaptations and adherence (50, 51). MIIT can also offer an advantage and reach a broader population by encouraging those averse to high-intensity exercises, such as some physically inactive and unfit individuals (50).

In a study evaluating body composition, resting blood pressure, heart rate (HR), and functional performance in elderly women, subjects underwent MIIT similar to HIIT but at moderate intensity to discern if HIIT benefits stemmed from its intermittency or intensity (52). The findings indicate that eight weeks of MIIT produced slightly better body composition results than MICT but were statistically lower than HIIT, emphasizing intensity's significance for this outcome. However, the effects of intermittency can't be dismissed, as MIIT surpassed MICT in some areas, supporting prior claims about its potential benefits (50). Although not extensively studied, intermittent and continuous exercises have shown different physiological responses despite similar levels of energy expenditure and intensity. Intermittent exercise may particularly favor mitochondrial development by possibly increasing the recruitment of fast-twitch fibers and inducing more pronounced metabolic variations (53).

Notably, interval training's complexity is underscored by controversies, impeding the ability to reach general conclusions (54). To support researchers and health professionals, experimental studies should provide detailed methodological reports, while future reviews and meta-analyses need to critically evaluate included articles' methods to prevent unwarranted generalizations (54). Additionally, more research is needed to understand MIIT's role in performance and health, and to compare its effects with HIIT and MICT in similar populations, elucidating which benefits can be attributed to changes in training parameters like intensity or stimulus type (50).

Other evidence also highlighted the importance of exercise intensity component in various other relevant health outcomes, as exemplified below. In a recent systematic review and meta-analysis on the dose-response relationship between PA levels and glycemic control in type 2 diabetes, it was found that individuals with diabetes may benefit from higher-than-recommended physical activity levels for optimal health outcomes. The optimum activity dose was 1,100 MET min per week, regardless of the baseline level of glycemic control. On average, this equals 244 min of moderate-intensity or 157 min of vigorous-intensity endurance activity per week (55). In a different systematic review and meta-analysis that assessed 25 pertinent studies, it was reported that both HIIT and MICT yield comparable effects on the quality of life and mental health of individuals with CVD. However, HIIT stands out as particularly advantageous for improving patients' self-perceived physiological functioning, taking into account their health status and social adjustment, especially among those with CAD (56).

Noteworthy, certain essential aspects of CR, including those pertaining to the intensity of endurance exercise, have not been entirely standardized yet (13), given the considerable variation in guidelines globally regarding recommendations (Table 1).

**Table 1:** Aerobic exercise intensity prescription across different guidelines and methods.

	Clinical Guideline	Cardiopulmonary exercise test	Exercise test without gas exchange analysis	Other functional tests	Rating of perceived exertion	Talk test
Australia and New Zealand	2023. A Clinical Guide for Assessment and Prescription of Exercise and Physical Activity in Cardiac Rehabilitation. A Cardiac Society of Australia and New Zealand Position Statement (57)	%VO_2peak_: Light: <40% Moderate: 40%–69% High: 70%–85% Very high: >85%	%HR_peak_: Light: <55% Moderate: 55%–74% High: 75%–90% Very high: >90%	Cited (evaluation): Incremental Shuttle Walk Test 6MWT	Borg: Light: 10–11 Moderate: 12–13 High: 14–16 Very high: 17–19	Light: Able to sing. Moderate: Able to talk in full sentences. High: Unable to talk comfortably.
			%HRR: Light: <40% Moderate: 40%–69% High: 70%–85% Very high: >85	6MWT average speed: Moderate: 80% High: 100%	Borg Modified: Light: 2–3 Moderate: 4–6 High: 7–8 Very high: 9	
Canada	2017. Comprehensive Update of the Canadian Cardiovascular Society Guidelines for the Management of Heart Failure (58)	%VO_2peak_: Moderate: 50%–75%	%HR_peak_: Moderate: 65%–85%	Cited (evaluation): 6MWT Intensity prescription not specified	Borg: Moderate: 3–5	Not specified
Europe	2021. ESC Guidelines on Sports Cardiology and Exercise in Patients With Cardiovascular Disease (59)	%VO_2peak_: Low: <40% Moderate: 40%–69% High: 70%–85% Very high: >85%	%HR_peak_: Low: <55% Moderate: 55%–74% High: 75%–90% Very high:>90%	Not specified	Borg: Low: 10–11 Moderate: 12–13 High: 14–16 Very high: 17–19	Cited (exercise monitoring possibility). Intensity prescription not specified
		Optimal intensity for patients with chronic HF: %VO_2peak_: 40%–80% (interval or continuos)	%HRR: Low: <40% Moderate: 40%–69% High: 70%–85% Very high: >85%			
	2022. Exercise intensity assessment and prescription in cardiovascular rehabilitation and beyond: why and how: a position statement from the Secondary Prevention and Rehabilitation Section of the European Association of Preventive Cardiology (6)	VTs: Low: <VT_1_ Moderate: between VT_1_ and VT_2_ High: >VT_2_	%HR_peak_: Low: <55% Moderate: 55%–74% High: 75%–90% Very high:>90%	Not specified	Borg: Low: 10–11 Moderate: 12–13 High: 14–16 Very high: 17–19	Cited as an adjunct practical way to guide intensity Moderate: maintain a level of exercise while still being able to talk comfortably in full sentences.
			%HRR: Low: <40% Moderate: 40%–69% High: 70%–85% Very high: >85%			
	2012. French Society of Cardiology Guidelines for Cardiac Rehabilitation in Adults (60)	Prescription intensity in steady-state endurance training: HR at VT_1_	%HRR: 60% if patient is without beta-blockers 80% if patient is taking beta-blockers	Cited (evaluation): 6MWT	Borg: 12–14	Able to speak easily without becoming breathless
			If angina: HR <10 bpm under the threshold for angina	Intensity prescription not specified	Borg Modified: 4–6	
	2008. Position paper of the Belgian Working Group on Cardiovascular Prevention and Rehabilitation: Cardiovascular Rehabilitation (61)	%VO_2peak_: 45%–85% Interval training is indicated as possibly more effective than continuous aerobic exercise training	%HR_peak_: 60%–90%	Not specified	Not specified, but cited	Not specified
	2013. Austrian Cardiac Society: Outpatient Cardiac Rehabilitation: the Austrian model (62)	80%–90% of the HR at the VT_1_	50%–70% of HR_peak_ during ergometry.	Not specified	Not specified	Not specified
	2011. Royal Dutch Society for Physical Therapy KNGF Clinical Practice Guideline for Physical Therapy (63)	%VO_2peak_: Very low: <25% Low: 25%–44% Moderate: 45%–59% High: 60%–84% Very high: ≥85% Maximum: 100%	%HR_peak_: Very low: <30 Low: 30–49 Moderate: 50–69 High: 70–90 Very high: ≥90 Maximum: 100	Cited (evaluation and screening): SWT 6MWT	Borg: Very low: <9 Low: 9–10 Moderate: 11–12 High: 13–16 Very high: >16 Maximum 20	Not specified
		The aerobic endurance or interval training level can gradually be increased from 50% to 80% of VO_2_max	%HRR: Very low: <25% Low: 25%–44% Moderate: 45%–59% High: 60%–84% Very high: ≥85% Maximum 100%			
		HIIT is indicated as possibly more effective than moderate-intensity endurance training.	Reported: absolute intensity (in METs) by age.			
International	2016. Cardiac Rehabilitation Delivery Model for Low-Resource Settings: An International Council of Cardiovascular Prevention and Rehabilitation Consensus Statement (64)	Low-risk patients: light to moderate-intensity	Low-risk patients, light to moderate-intensity	Cited (evaluation): 6MWT Step test	Not specified, but cited	Not specified
		Not specified	Not specified	Not specified		
	2016. Cardiac Rehabilitation Delivery Model for Low-resource Settings (Endorsed by: International Council of Cardiovascular Prevention and Rehabilitation) (65)	Not specified	If an exercise ECG has been conducted: HR during exercise should be kept below the symptomatic threshold	Cited (alternative evaluation): 6MWT	Borg: 11–16	Not specified
				Intensity prescription not specified		
Japan	2022. JCS/JACR 2021 Guideline on Rehabilitation in Patients with Cardiovascular Disease (66)	At VT_1_	General recommendation %HRR: 40%–60% 60% - young patients with uncomplicated AMI 40%–50% for high-risk patients 30%–50% for HF patients.	Cited (evaluation): 6MWT SWT	Borg: 12–13	Intensity that can be done while talking comfortably
		Patients with acute myocardial infarction after the recovery phase: HR at VT_1_ or 40%–60% of VO_2peak_.				
		HIIT may be considered in the maintenance period and should reach at least 80% of maximum intensity.	%HR_peak_: High: 85%–95% Moderate: 60%–70%	Intensity prescription not specified	Borg: 11–13 for HF patients	
Korea	2019. Clinical Practice Guideline for Cardiac Rehabilitation in Korea (67)	Not specified	Not specified	Cited (alternative evaluation): 6MWT	Not specified	Not specified
		Recommended including aerobic exercise in CR programs; HIIT may be more effective than aerobic exercise		6MWT distance is used to set intensity		
South America	2020. Brazilian Cardiovascular Rehabilitation Guideline (14)	VTs: Low <VT_1_ Moderate: between VT_1_ and VT_2_ High >VT_2_	%HR_peak_: Moderate: 70%–85%	Cited (alternative evaluation): 6MWT Step test	Borg: Moderate: 10–13	Moderate: maintain exercise intensity for controlled yet labored breathing, allowing the patient to speak complete sentences without interruption
			%HRR: Moderate: 50%–80%	Intensity prescription not specified	Borg Modified: Moderate: 2–4	
	2014. South American Guidelines for Cardiovascular Disease Prevention and Rehabilitation (68)	Phase 2: around VT_1_	Phase 2: %HR_peak_: 60%–80% %HRR: 50%–70%	Cited (alternative evaluation): 6MWT	Borg: Should always be assessed	Exercise at an intensity causing heavier breathing without reaching a level of tachypnea hindering sentence completion
		Phase 3 and 4: between VT_1_ and VT_2_	Phases 3 and 4 (asymptomatic patients) %HR_peak_: 70%–90% %HRR: 50%–80%	Intensity prescription not specified	Intensity prescription not specified	
UK	2023. Association of Chartered Physiotherapists in Cardiac Rehabilitation Standards: Standards for Physical Activity and Exercise in Cardiovascular Population (69)	Minimum training intensity: VT_1_	%HRR: 40%–70%	Cited (functional capacity measures): 6MWT Incremental SWT Chester Step Test The Duke Activity Status Index	Borg: 11–14	Not specified
		%VO_2peak_: Moderate: 40%–70%	Lower intensities (30%–50% HRR) for significantly deconditioned individuals			
		Consider short bouts of high and low intensity exercise				
		HITT: >85% peak power output or VO_2peak_	Higher levels of fitness may require a training intensity at the upper limit of 70%HRR			
	2013. Irish Association of Cardiac Rehabilitation: Cardiac Rehabilitation Guidelines (70)	VO_2peak_: 40%–80%	%HR_peak_: 50%–85%	Cited (alternative evaluation): 6MWT Chester Step Test Modified SWT	Borg: 13–16	Not specified
			%HRR: 40%–70%	Intensity prescription not specified	Borg Modified: 2.5–6	
USA	American College of Sports Medicine. Guidelines for Exercise Testing and Prescription (71)	%VO_2_ Reserve: Very light: <30 Light: 30%–39% Moderate: 40%–59% Vigorous 60%–89% Near maximal to maximal: ≥90%	%HRR: Very light: <30 Light: 30%–39% Moderate: 40%–59% Vigorous: 60%–89% Near maximal to maximal: ≥90%	Cited (evaluation): 6MWT SWT	Borg: Very light: <9 Very light to fairy light: 9–11 Fairly light to somewhat hard: 12–13 Somewhat hard to very hard: 14–17 ≥Very hard: ≥18	Not specified
		%VO_2peak_: Very light: 37% Light: 37%–45% Moderate: 46%–63% Vigorous: 64%–90% Near maximal to maximal: ≥91%	%HR_peak_: Very light: 57% Light: 57%–63% Moderate: 64%–76% Vigorous 77%–95% Near maximal to maximal: ≥96%	Intensity prescription not specified		
			Reported: intensity in METs by age.			

AMI, acute myocardial infarction; CR, cardiac rehabilitation; HIIT, high intensity interval training; HF, heart failure; SWT, shuttle walk test; VT_1_, first ventilatory threshold; VT_2_, second ventilatory threshold; %VO_2peak_, percentage of peak oxygen uptake; %HR_peak_, percentage of peak heart rate; %VO_2_Reserve; 6MWT, six-minute walk test.

## 3 Assessing patients at risk for or with CVD: methods for tailoring endurance exercise intensity prescription

An appropriate endurance exercise intensity prescription depends on an adequate assessment of the patient (6) and may consider the available resources in different settings to optimize exercise interventions (64, 65).

### 3.1 Cardiopulmonary exercise test (CPET)

The CPET is considered the gold standard assessment for exercise intensity prescription, and the threshold-based methodology should be the primary choice (5, 6, 14, 72–74) because it incorporates parameters that offer a comprehensive description of the body's response to exercise in terms of oxygen transport and utilization (13). Hence, this approach is recognized for accurately quantifying intensity and for being more tailored to individual phenotype (5, 6, 14, 72–74). This concept is grounded in physiological principles, as individual differences in transitioning from aerobic to anaerobic metabolism are associated with clinical factors such as prior interventions, medication use, and habitual physical activity levels, as well as with abnormalities in ventilation, cardiovascular function, or muscle physiology (73, 75).

The first ventilatory threshold (VT_1_) signifies the point at which there is a transition from predominantly aerobic metabolism to a stage where blood lactate begins to increase but ultimately reaches a balance. This balance is achieved through an equal rate of lactate production and consumption as a reactant in metabolic chemical reactions, thereby maintaining lactate levels within the range of 1.5–2 mmol/L (6). The second ventilatory threshold (VT_2_), also known as the respiratory compensation point, is the intensity at which blood lactate rapidly increases. Lactate accumulation thus emerges due to tissue anaerobiosis, leading to a disproportionate increase in minute ventilation (VE) relative to carbon dioxide production (VCO_2_), with lactate levels between 3 and 5 mmol/L (6).

In contrast to peak exercise capacity indices, both VT_1_ and VT_2_ are achievable by most CVD patients and are not dependent on maximum effort (6). So, it is recommended to prescribe the exercise intensity based on VTs, extrapolated to HR, load, or time (6, 14, 60, 62, 66, 68, 69).

While it is widely recommended to base exercise prescriptions on %VO_2peak_, which is directly measured during CPET, it is crucial to note that relying on fixed percentages of VO_2peak_ may not be optimal. This approach fails to consider individual variations in fitness, body mass, age, and sex, all of which significantly influence cardiac and metabolic demands, particularly in low fitness levels (76), leading to varied and unexpected cardiac and metabolic demands (77). For example, Milani et al. (78) showed that in CVD patients the VT_1_ varied from 52% to 65% of VO_2peak_, and VT_2_ from 85% to 93% of VO_2peak_, which are considerable wide ranges.

While CPET is the optimal standard for prescribing endurance exercise, it is costly and may not always be accessible before CR. Furthermore, VTs estimation can be inaccurate, affected by inter-observer variability, and sometimes impractical for assessment, particularly in highly deconditioned individuals and those with HF (13).

### 3.2 Exercise test without Gas exchange analysis

According to the official position statement from the Secondary Prevention and Rehabilitation Section of the European Association of Preventive Cardiology regarding exercise intensity assessment and prescription in CR (6), performing an exercise test without gas exchange analysis is considered the minimum standard for accurately prescribing endurance exercise. This exam is commonly used in clinical practice because it is easy to perform and cost-effective, yielding measurements such as peak heart rate (HR_peak_), peak workload (W_peak_), and MET_peak_ that have been utilized for years to tailor exercise intensity. Most CR guidelines recommend that exercise intensity should then be based on peak effort indicators, such as percentages of peak workload (%W_peak_) and %HR_peak_, or heart rate reserve (%HRR) (79). Therefore, it is crucial to explicitly define the correlation of these indices with the established preferable standards, the VT_1_ and VT_2_ direct measure method (13). Substantial differences in individual physiological responses at the VTs, such as HR or workload, and their alignment with widely used guideline-directed exercise intensity domains have been noted (72, 78, 80–85). This is important, as improperly setting exercise intensities may lead to under- or overtraining and can impact patient motivation and safety (6, 72, 78, 80–82, 84).

Importantly, the training status notably affects the relationship between VT_1_ or VT_2_ and exercise intensity domains. For instance, Hansen et al. (80), comparing physically unfit CVD patients (VO_2peak_ < 15 ml/min/kg) to fit ones (VO_2peak_ > 25 ml/min/kg) revealed significantly different exercise intensity domains for VT_1_ and VT_2_. Therefore, clinicians should exercise caution when applying guideline-recommended percentages of exercise intensity levels, considering changes in CRF during CR (80).

Furthermore, it is important to consider other cardiovascular conditions that may also influence exercise intensity prescription, including the presence of an ischemic threshold identified during exercise testing and its clinical consequences such as angina, arrhythmias, and hypotension (86). Additionally, symptoms like dyspnea or angina leading to test termination, slow HR recovery 1 min after the test, and abnormal hemodynamic and/or electrocardiographic responses provide additional evidence of poor prognosis and increased severity (87). It is also crucial to consider peripheral arterial disease, which requires evaluating the lower limb claudication threshold on a treadmill. It's worth noting that exercise testing performed on a cycle-ergometer allows for more precise cardiopulmonary evaluation in these cases (86).

Given the limitations highlighted regarding HR-based approaches and the incomplete representation of CRF by W_peak_ in CVD patients (13), it is recommended to incorporate additional subjective methods guided by health professionals, such as the “talk test” (TT) and Borg Ratings of Perceived Exertion (RPE), for monitoring and adjusting exercise intensity during sessions (6).

### 3.3 Other functional tests (submaximal tests)

According to the International Council of Cardiovascular Prevention and Rehabilitation Consensus (64), conducting exercise assessments, such as field-based tests like walking or step-up protocols, is essential even in settings with limited resources. Hence, it is recognized that all CR programs have the capability to conduct at least some objective physical assessment of physiological responses to exertion, which is considered the minimum requirement.

Consequently, functional tests are essential, especially when exercise tests with or without gas exchange analyses are unavailable or unfeasible. Functional tests assist in clinical assessment, provide criteria for comparing functional capacity during training, and obtain accurate individual physiological parameters during physical exertion to guide exercise prescription (14, 88–91).

The 6-minute walk test (6MWT) is the most recommended submaximal functional assessment according to current guidelines, followed by the Shuttle–Walk Test (SWT) and Step Test (ST) (Table 1).

The 6MWT is documented as a valid and reliable field test, reflecting the performance of daily life activities (90, 91). It is an uncomplicated assessment that requires no specialized equipment or advanced training. The test evaluates an individual's submaximal functional capacity according to the maximum distance covered as they walk on a flat, hard surface for 6 min. Additionally, it collects information on the physiological responses such as blood pressure, HR, pulse-oximetry, and the patient's self-assessment of fatigue and dyspnea (90).

In patients with HF the 6MWT is a safe and low-cost alternative for the prescription of endurance exercise (92), with prognostic value comparable to VO_2peak_ (93–95). However, caution should be applied when prescribing endurance exercise based on HR_peak_ in the 6MWT. Some studies indicate a correlation with VT_1_ (89, 96), while other authors report that HR_peak_ in the exam closely resembles HR at VT_2_ (97). Importantly, the studies in the first situation used HR data from the final minute of the 6MWT, while in the second reported situation, HR_peak_ was identified considering the entire test.

Oliveira et al. (89) reported that exercise prescription for patients with HFrEF can be determined using the combination of the 6MWT and ST, relying on the HR values obtained during these tests. A robust correlation was observed between HR at VT_1_ and 6MWT HR (*r* = 0.81; *p* < 0.0001) as well as between HR_peak_ and ST HR (*r* = 0.89; *p* < 0.0001). Additionally, the authors proposed two types of exercise prescriptions to achieve the ideal HR target using these tests: (i) HR_6MWT_ + 10%, or (ii) HR_6MWT_ to HR_ST_ − 10% (both associated with Borg RPE scale). Previous research also reported the robust correlation between ST HR and HR_peak_ (98).

SWT is also a functional test option reported in CR guidelines (57, 66, 71), which is characterized by incremental intensity and external pacing (99). This test has proven to be reliable (100, 101), and it is employed in diverse clinical populations, encompassing individuals with CVD (102). Incorporating the SWT into the initial stages of an exercise training program is considered a valid option for tailoring the appropriate exercise intensity and functions as an outcome measure to assess the program's effectiveness (103). Nevertheless, there is also a notable absence of specific, objective recommendations on current guidelines concerning exercise intensity prescription based on these submaximal tests (Table 1).

### 3.4 Borg ratings of perceived exertion and talk test

Exercise intensity prescription can be guided by utilizing subjective assessments such as Borg scales or the TT (6, 73, 104). These tools are particularly valuable for overseeing the progression of exercise intensity in MICT programs, especially in home-based settings (6), and are strongly endorsed in current guidelines (Table 1).

In specific clinical groups, such as individuals on beta-blockers with a limited chronotropic reserve, monitoring HR becomes less advantageous (104). Moreover, training responses resulting from subjective methods closely mirror those prescribed through traditional methods (105, 106), suggesting that in these cases, subjective methods could even serve as a primary means for determining intensity (107).

A recent study by Bok et al. (104) confirmed the reliability and validity of both tools in defining ventilatory and lactate thresholds, stating that these measures can efficiently induce homeostatic disturbances associated with moderate, heavy, and severe-intensity domains during continuous exercise.

Consequently, when peak exercise data are unavailable or when HR is inaccessible or inapplicable (e.g., atrial fibrillation, pacemaker usage, or chronotropic incompetence) the RPE scale is as a valid alternative, despite its variability (108). Patient-reported RPE using the Borg scale promotes patient autonomy, allowing clinicians to steer individuals into specific intensity ranges, and is widely integrated into clinical practice (73). Remarkably, a more positive affective response was noted when individuals chose their own exercise intensities (109).

However, it's important to note that patients who report RPE may be affected by factors beyond the physical effort of the exercise, including psychological elements and environmental conditions. Furthermore, challenges in interpreting RPE may arise due to factors such as lack of familiarity with exercise training (modes/equipment), lower educational levels, and the use of beta-blockers (73).

In a study involving 2,560 participants of various ages, sexes, and fitness levels, incremental exercise tests revealed correlations between RPE and lactate levels (*R*^2^ = 0.71) and between RPE and HR (*R*^2^ = 0.55). The mean and standard deviation of RPE values (on Borg's scale of 6–20) for VT_1_ and VT_2_ were respectively 10.8 ± 1.8 and 13.6 ± 1.8 (110). Thus, for clinical application, an Borg RPE around 10–11 may reflect VT_1_, while values around 13–14 may correspond to VT_2_ (104).

However, caution is advised for healthcare professionals when prescribing moderate to high-intensity workouts to individuals with health complications based solely on RPE assessments, as it is also reported that the reliable agreement between RPE and blood lactate levels concentrations is demonstrated only during low-intensity exercises (111).

Similar to the Borg RPE, the TT can be employed as a supplementary practical method to manage exercise intensity in the daily activities of CVD patients (6, 112), delineating specific training zones during endurance exercise assessments and prescription (97, 113). This simple yet reasonably accurate method uses the comfort level of speech perception as a gauge for exercise intensity, allowing individuals to easily assess the intensity zone of their exercise (113, 114).

Numerous studies have validated the TT as a reliable method for estimating the lactate and VTs across diverse populations, including individuals with CVD (97, 115, 116), overweight and obese individuals (117). The observed parallels between HR and VO_2_ at TT stages and VTs in individuals with CVD suggest the potential application of TT stages in prescribing endurance exercise (97, 118). Importantly, it has been demonstrated that ischemic patients who can speak comfortably are unlikely to exhibit ECG evidence of myocardial ischemia (119). Moreover, it exhibits a close correlation with RPE (120).

Recently, Althoff et al. (97) highlighted that TT-positive (comfortable speech still possible during exercise) and TT-negative (patient unable to read the paragraph comfortably) are useful instruments to prescribe endurance exercise to patients with CVD. These tools exhibited a moderate relationship with HR at VT_1_ (*r* = 0.479) and a strong relationship with HR at VT_2_ (*r* = 0.757), respectively. These results are aligned with others (104, 121).

In summary, the TT's equivocal stage, characterized by somewhat uncomfortable speech, corresponds to intensity above VT_1_, while the negative stage, where a person is no longer able to recite the paragraph comfortably, aligns with intensity above VT_2_ (104).

**Figure 1:**
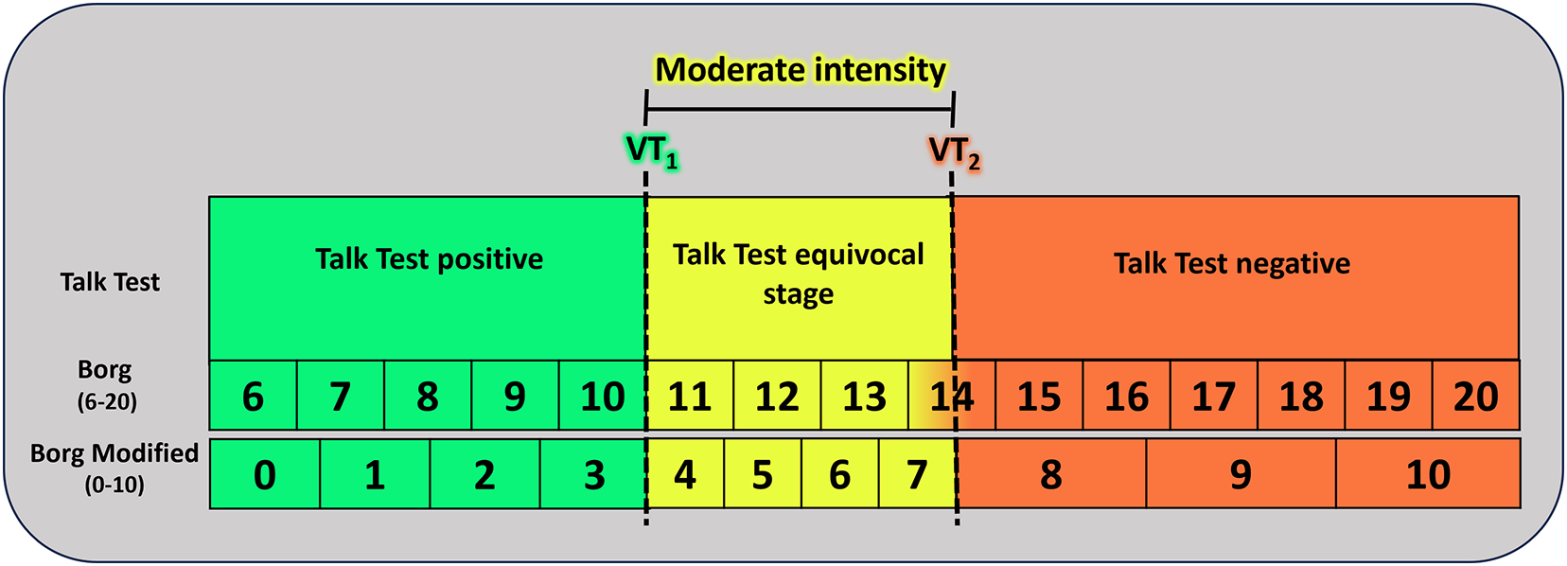
Practical guidance for monitoring exercise intensity using the talk test and the rating of perceived exertion scale. Adapted from: Bok et al. (104) and Festa et al. (113). VT_1_, first ventilatory threshold; VT_2_, second ventilatory threshold. Talk Test positive: comfortable speech still possible during exercise; Talk Test equivocal stage: characterized by somewhat uncomfortable speech; Talk Test negative: patient unable to read the paragraph comfortably.

Figure 1 provides a practical guidance on utilizing the TT and the RPE scale to monitor exercise intensities.

### 3.5 Predictive equations for HR_peak_

Prediction equations for HR_peak_ are also used in CR contexts (122–125), although prescribing exercises based on the predicted values of HR_peak_ can be quite divergent, especially in patients with CVD (14, 78, 126, 127), mainly due to negative chronotropic influence of the beta-blockers, and disease-related dysfunctions (14). Although recently recommended in a guideline (69), it is important to emphasize that even equations for estimating HR_peak_ developed in samples of patients with CVD show significant dispersion of individual responses (124–126) and are not based on individual physiological responses to physical effort, but rather on age and resting parameters, such as resting HR (HR_rest_) (124, 125), ejection fraction, and hemoglobin level (125). Mytinger et al. (127) reported that HR_peak_ estimation equations are outdated for these patients and should not be used as frequently as done by healthy populations.

The increased dispersion between the actual individual HR_peak_ measurements and the predicted values may result in significant inaccuracies in the identification of exercise prescription domains (126). Therefore, considering evidence that intensity domains prescribed as a percentage of measured HR_peak_ can be highly inconsistent with guideline-based recommendations due to individual variability in HR response to exercise (72, 78, 80–85), determining exercise intensity based on estimated values may result in even greater inconsistencies. Hence, this approach could compromise the accuracy of CR prescriptions, potentially affecting efficacy and safety (126).

In this context, even in low-resource settings, it is advisable for the exercise intensity prescription to always depend on an objective assessment of individual physiological variables during effort. Therefore, at least a submaximal functional test should always be performed, along with subjective methods such as Borg RPE and/or TT, to adjust the prescription.

## 4 International standards for exercise intensity prescription: a comprehensive overview of CR guidelines

As previously stated, structured exercise training, aimed at enhancing physical fitness and prognosis, stands as a cornerstone in contemporary comprehensive CR (2–4, 128–133). The guidelines provide evidence-based best practice recommendations for CR, reducing disparities in the implementation treatments and in the development of a universal, evidence-based CR program (67).

However, the quantification of exercise training frequency, duration, and particularly intensity differs across current international CR guidelines (79). Numerous guidelines, position statements, and policy documents worldwide have proposed various thresholds for assessing exercise intensity prescription of an exercise-based CR program (79) (Table 1).

The majority of CR guidelines advise determining endurance exercise training intensity by referencing peak effort indices (such as %VO_2peak_ and %HR_peak_) or %HRR. Nonetheless, using peak exercise capacity indices for exercise prescription in CVD patients is challenging. Approximately 15% of general outpatient CR patients (80) and 46% of HF patients (82) do not achieve maximal effort (RER > 1.10) during CPET. In addition, a plateau in VO_2_ is often not observed (80, 134), and different peak indices are not interchangeable for exercise training prescription (6).

Moreover, the association with subjective methods, Borg and/or the TT, is widely reported. Importantly, the International Council of Cardiovascular Prevention and Rehabilitation Consensus (64) emphasizes the necessity of conducting exercise assessments, even in resource-constrained settings, which may include the use of field-based tests such as walking or step-up protocols. Regarding these functional tests, the 6MWT is the most cited, but most of the documents do not provide specific instructions about intensity prescription determination (Table 1).

It is important to highlight that the patients undergoing CR exhibit significant inconsistencies between guidelines and individual threshold-based intensities (72, 78, 80–85). These observations highlight that strict adherence to guideline-based exercise intensity domains can lead to erroneous prescriptions, especially in challenging situations, such as in patients with low fitness (80). In addition, the guidelines notably delineate exercise intensity domains validated in healthy individuals, who's cardiopulmonary and muscle physiology significantly differs from that of CVD patients (135). These disagreements have sparked discussions about reevaluating the necessity of reconsidering the current guidelines for exercise prescription in the CR setting (13, 80).

Therefore, the optimal approach to avoid relying on the mentioned peak exercise intensity indices is to associate training intensity with VT_1_ and VT_2_, which reflect real metabolic responses to submaximal effort in the moderate-intensity domain, are not dependent on maximum effort and can be attained by the majority of CVD individuals (6, 14).

Unfortunately, the direct VTs assessment by CPET is not the reality for many CR services (136). Krieger et al. (136), for instance, found that 50% of 91 American rehabilitation programs used submaximal tests, such as the 6MWT, while only 2% conducted incremental exercise testing. This scenario may be even worse in lower-income countries. As earlier reported, in lower-resource settings, the optimal strategy involves approximating VTs by integrating alternative objective assessments (ideally an ergometry test, or alternatively, another functional test providing individual physiological effort parameters like the 6MWT) alongside subjective methods for guiding prescription adjustments, such as the Borg scale and the Talk Test.

## 5 Integrating the “Intensity” component within the FITT-VP exercise prescription model

As previously mentioned, the planning and implementation of exercise prescription in CVD should be aligned with the FITT-VP model [F: frequency, I: intensity, T: duration (time), T: type of specific exercise, V: volume, P: progression] (16, 17). In this context, intensity emerges as the most complex component, triggering significant controversies over the reliability and validity of methods used to determine and prescribe training intensity (82, 137). Conversely, frequency, duration, and volume of training can be adjusted by varying session frequency, session duration, or total workload within a specific timeframe (82, 137). Furthermore, as previously detailed, notable inconsistencies and disagreements exist among recommendations from different CR guidelines (6, 78, 80, 84). These factors can often result in suboptimal exercise prescriptions for many individuals and limit the ability to compare outcomes across various training programs in clinical practice and research (13, 78, 82).

A general exercise recommendation for both primary and secondary prevention of CVD includes at least 150 min of light to moderate intensity endurance exercise per week, spread over 3–5 days, along with moderate-intensity dynamic resistance exercises on 2 days (6, 7, 14). While this initial guidance is suitable for starting physical activity, adapting the prescription based on individual conditions, risk profile, and needs is crucial to maximize exercise's clinical benefits and prevent CVD (7). This approach is supported by documented additional clinical benefits from aligning prescription components with specific risk factors (7).

**Figure 2:**
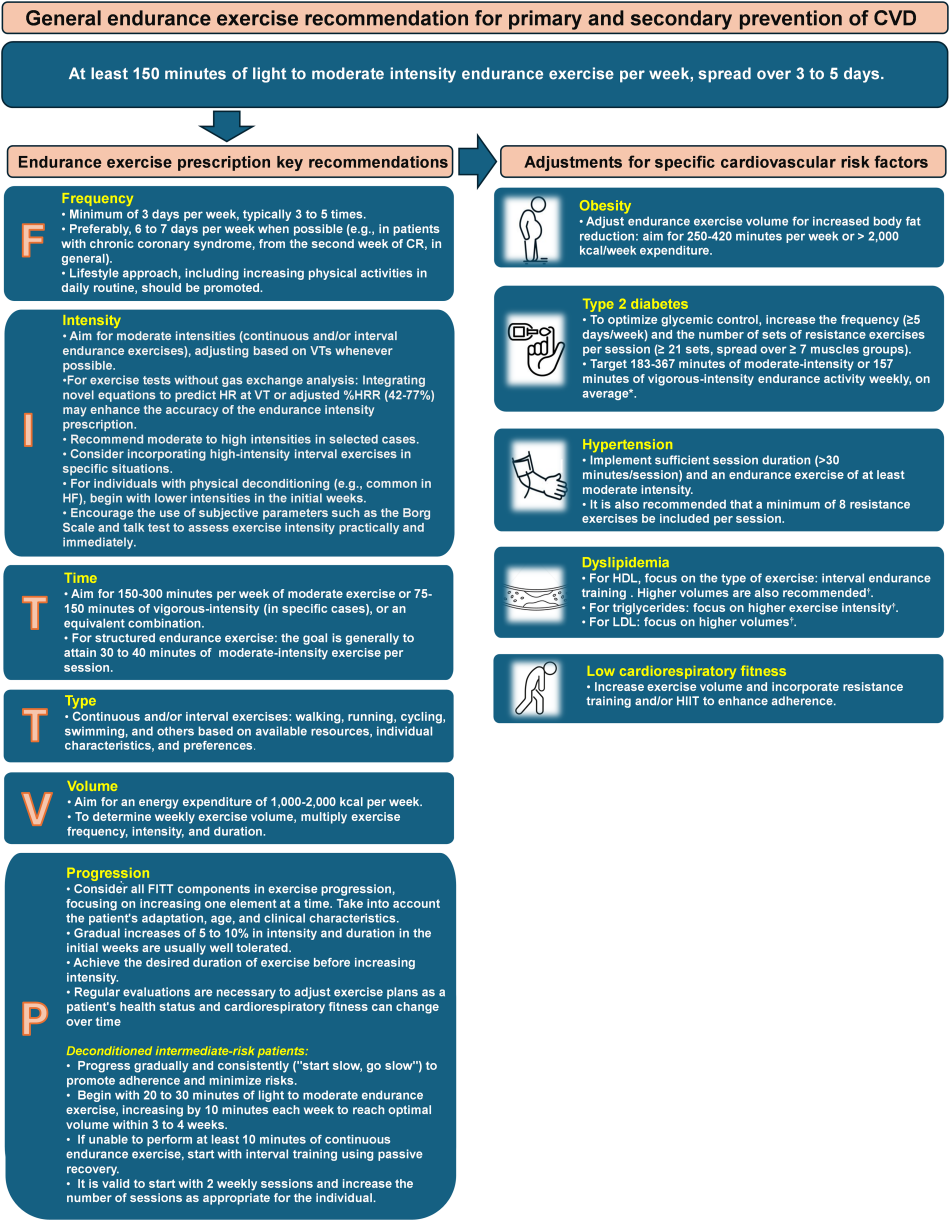
Key recommendations for endurance exercise prescription in cardiovascular rehabilitation patients. Adapted from: D'Ascenzi et al. (5), Hansen et al. (6, 7, 21). *Gallardo-Gomez et al. (55). ^†^Wood el al. (29). CR, cardiovascular rehabilitation; CVD, cardiovascular diseases; HDL, High-Density Lipoprotein; HF, heart failure; HIIT, high intensity interval training; VTs, ventilatory thresholds.

Considering the FITT-VP principles, Figure 2 outlines key recommendations for a personalized endurance exercise prescription in CR (5–7, 21). Other exercise modalities, such as strength and flexibility training, should also be included in the program but are not covered in this article.

## 6 Equations to predict HR at VTs: a new perspective

In a recent study (78) involving 972 maximal treadmill CPET on patients with CVD, a novel approach was proposed, using multivariable equations to estimate HR at VTs, based on parameters individually assessed during an exercise test without gas exchange analyses (HR_peak_, HR_rest_, and MET_peak_), according to the Equations 1, 2.

(1) HRatVT1=3.453+(0.887×HRpeak)−(0.555×(HRpeak−HRrest))+(1.044×METpeak)

(2) HRatVT2=−8.256+(0.979×HRpeak)−(0.232×(HRpeak−HRrest))+(1.418×METpeak)

The equations demonstrated significant *R*^2^ for both VTs (0.726 for VT_1_ and 0.901 for VT_2_). Additionally, the method showed superior accuracy in internal validation, with a mean absolute percentage error (MAPE) of 6.0% for VT_1_ and 4.3% for VT_2_, compared to other guideline-based methods relying on measured and estimated %HR_peak_ (MAPE ranging from 9.5% to 23.8% for VT_1_ and 5.8% to 19.3% for VT_2_).

While this suggests their potential as a valid alternative when CPET is unavailable, additional studies are necessary to externally validate these treadmill equations for HR at VTs. Furthermore, since the equations were developed solely in the context of treadmill assessments, their applicability in cycle-ergometer exams remains untested.

However, equations to predict HR at VTs using cycle-ergometer assessments were recently developed in a European database of cardiometabolic disease patients and externally validated in a South American sample (138). These cycle-ergometer prediction equations, which also utilize variables measured during an exercise test without gas analysis, demonstrated excellent coefficients of determination, with R^2^ values of 0.77 for VT_1_ and 0.88 for VT_2_ (Equations 3, 4).

(3) HRatVT1=4.866+(0.405×HRpeak)+(0.542×HRrest)

(4) HRatVT2=−2.606+(0.773×HRpeak)+(0.254×HRrest)

In the external validation, these predictive equations for HR at VTs showed superiority over widely used guideline-directed intensity domains for %HR_peak_ and %HRR, exhibiting lower and more consistent MAPE values (VT_1_: 7.1%, VT_2_: 5.0%) in comparison to the range observed in the guideline-based exercise intensity domains (VT_1_: 6.8%–21.3%, VT_2_: 5.1%–16.7%) (138). Additionally, these novel equations showed ergometer interchangeability, as the validation sample included both treadmill and cycle-ergometer assessments.

Another important finding of this study was a suggestion to adjust the %HRR method, by using 42% for VT_1%_ and 77% for VT_2_ (138). These adjusted values for moderate intensity prescription demonstrated the same MAPE values of the predictive equations and were more consistent than the current guideline recommendations for %HRR.

In summary, assessing and prescribing endurance exercise intensity for patients with CVD involves various methods. The CPET stands as the gold standard, and utilizing VT_1_ and VT_2_ to tailor prescriptions based on individual physiological responses is the optimal approach to exercise prescription. Alternatives include simpler exercise tests without gas exchange analysis, offering less precision but practicality through measurements like HR_peak_ and workload. Importantly, novel equations to predict HR at these thresholds have been developed and validated, as well as a range-adjusted %HRR derived from CR patients, enhancing the accuracy of exercise intensity prescriptions when CPET is unavailable. Especially in resource-limited settings, submaximal functional tests like the 6-min walk test play a vital role, and subjective tools such as the Borg RPE and the Talk Test should always be applied as an addition to objective methods.

## 7 Intensity prescription in practice: clinical cases

The following clinical cases illustrate the prescription of endurance exercise intensity based on HR using different approaches, both with and without gas exchange analysis.

### 7.1 Clinical case 1

Male, 58 years. Weight: 76.3 kg, Height: 1.70 m, BMI: 26.4 kg/m^2^. Active smoker, dyslipidemia, family history of premature CAD.

Clinical History:

4 weeks before the first assessment in CR: acute event of anterior myocardial infarction with successful primary angioplasty (occlusion of the left anterior coronary artery—uniarterial disease).

• Medications: Antiplatelets, Metoprolol 50 mg/day, Ramipril 10 mg/day, and Rosuvastatin 20 mg/day.
• Echocardiogram with hypokinetic dysfunction in the anterior wall with a left ventricular ejection fraction of 45%.
• Treadmill CPET: Test interrupted by fatigue in lower limbs; Absence of myocardial ischemia; Maximal effort (RER_peak_: 1.30); Reduced CRF (VO_2peak_: 58% of predicted values—Wasserman); Peak treadmill load equivalent to 7.4 METs (FRIEND equation) (139).

**Figure 3:**
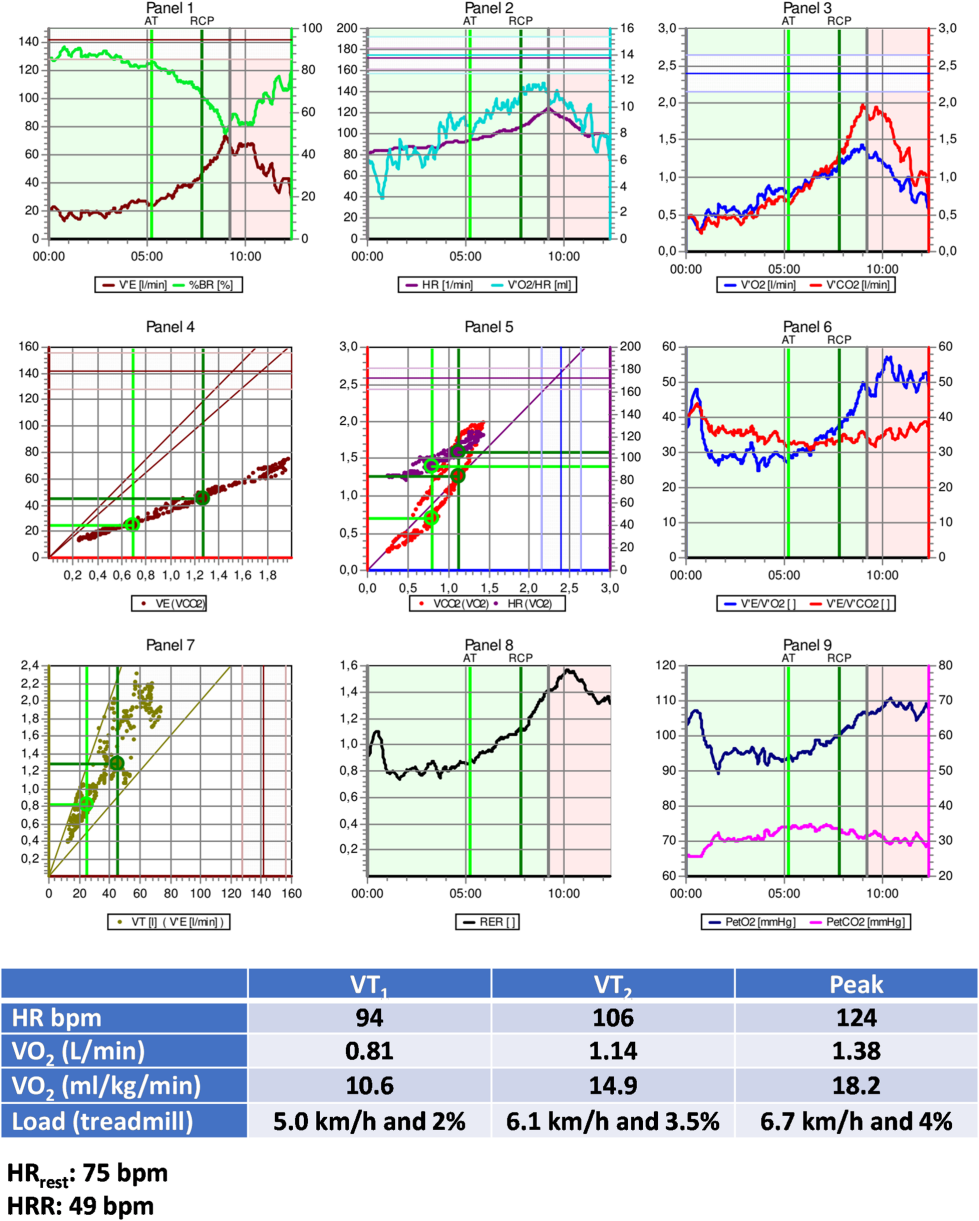
Exercise test information of clinical case 1. HR, heart rate; HRR, heart rate reserve; HR_rest_, rest heart rate; VO_2_, oxygen uptake; VT_1_, first ventilatory threshold; VT_2_, second ventilatory threshold.

**Figure 4:**
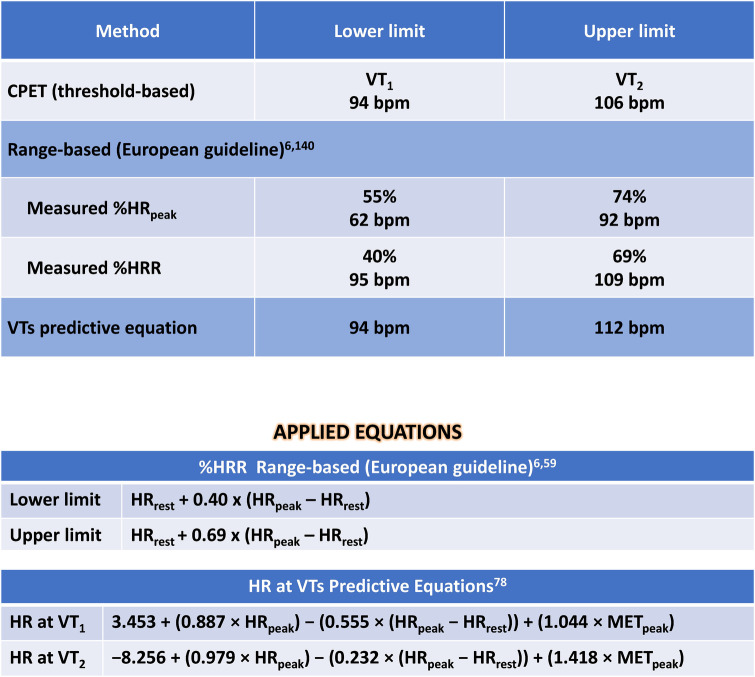
Solution of clinical case 1: prescribing moderate endurance exercise intensity using various heart rate-based approaches. CPET, cardiopulmonary exercise test; HR, heart rate; HR_peak_, peak heart rate; HRR, heart rate reserve; HR_rest_, rest heart rate; MET_peak_, peak metabolic equivalent of tasks; VO_2_, oxygen uptake; VTs, ventilatory thresholds; VT_1_, first ventilatory threshold; VT_2_, second ventilatory threshold; %HR_peak_, percentage of peak heart rate; %HRR, percentage oof heart rate reserve.

Figure 3 presents additional exercise test information, while Figure 4 illustrates the solution.

*Interpretation:*

The optimal method for exercise prescription involves utilizing the VTs identified during CPET, resulting in a moderate-intensity prescription of 94–106 bpm. It is evident that relying on %HR_peak_ can lead to a significant error in the prescription range: the upper limit guided by %HR_peak_ is, in fact, the lower limit based on threshold-based prescription. This discrepancy can result in under-training and likely diminish the benefits of exercise training based solely on HR_peak_.

Utilizing prescription guidance through %HRR or estimating HR at VTs using equations leads to range-based values similar to those identified by CPET. Therefore, a prescription based on two or more parameters (HR_rest_ and HR_peak_, with or without information about CRF) can be more individualized and, arguably, more beneficial.

### 7.2 Clinical case 2

Male, 62 years. Weight: 77.2 kg, Height: 1.78 m, BMI: 24.4 kg/m^2^. Former smoker, dyslipidaemia, diabetes mellitus, hypertension.

• Clinical history:

Chronic coronary artery disease with coronary artery bypass graft 10 years in the past.

• Medications: Antiplatelets, Metoprolol 75 mg/day, Ramipril 10 mg/day and Rosuvastatine/Ezetimiibe 20/10 mg/day, Metformine 2 g/day, Dapagliflozin 10 mg/day.
• Echocardiogram with hipocontratile disfunction in inferior wall with left ventricular ejection fraction of 40%.
• Cycle-ergometer CPET: Test interupted by dispnea; Absence of myocardial ischemia; Maximal effort (RER peak: 1.26); Reduced CRF (VO_2peak_: 71% of predicted values—Wasserman); Peak load equivalent to 6.3 MET (FRIEND equation) (140).

**Figure 5:**
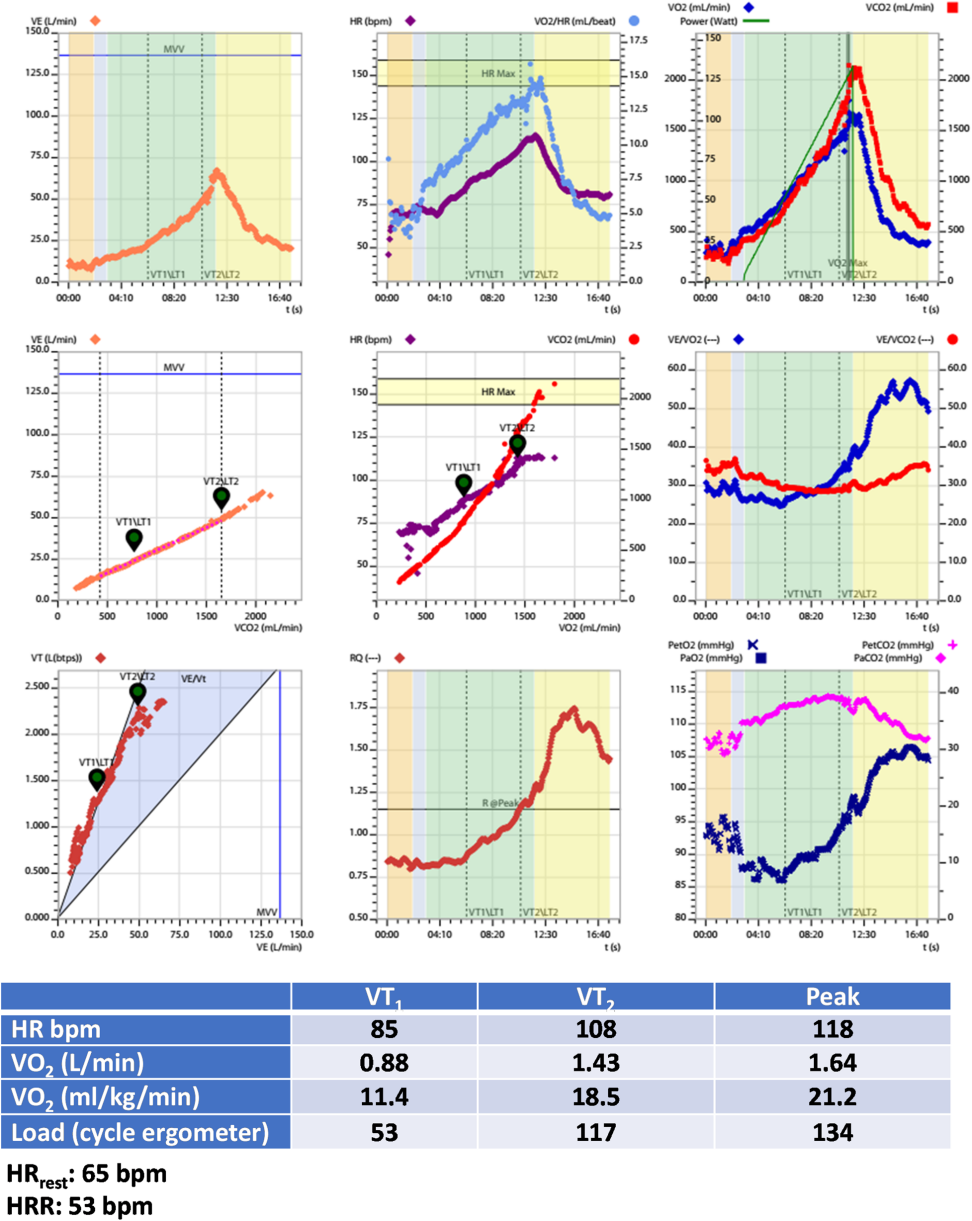
Exercise test information of clinical case 2. HR, heart rate; HRR, heart rate reserve; HR_rest_, rest heart rate; VO_2_, oxygen uptake; VT_1_, first ventilatory threshold; VT_2_, second ventilatory threshold.

**Figure 6:**
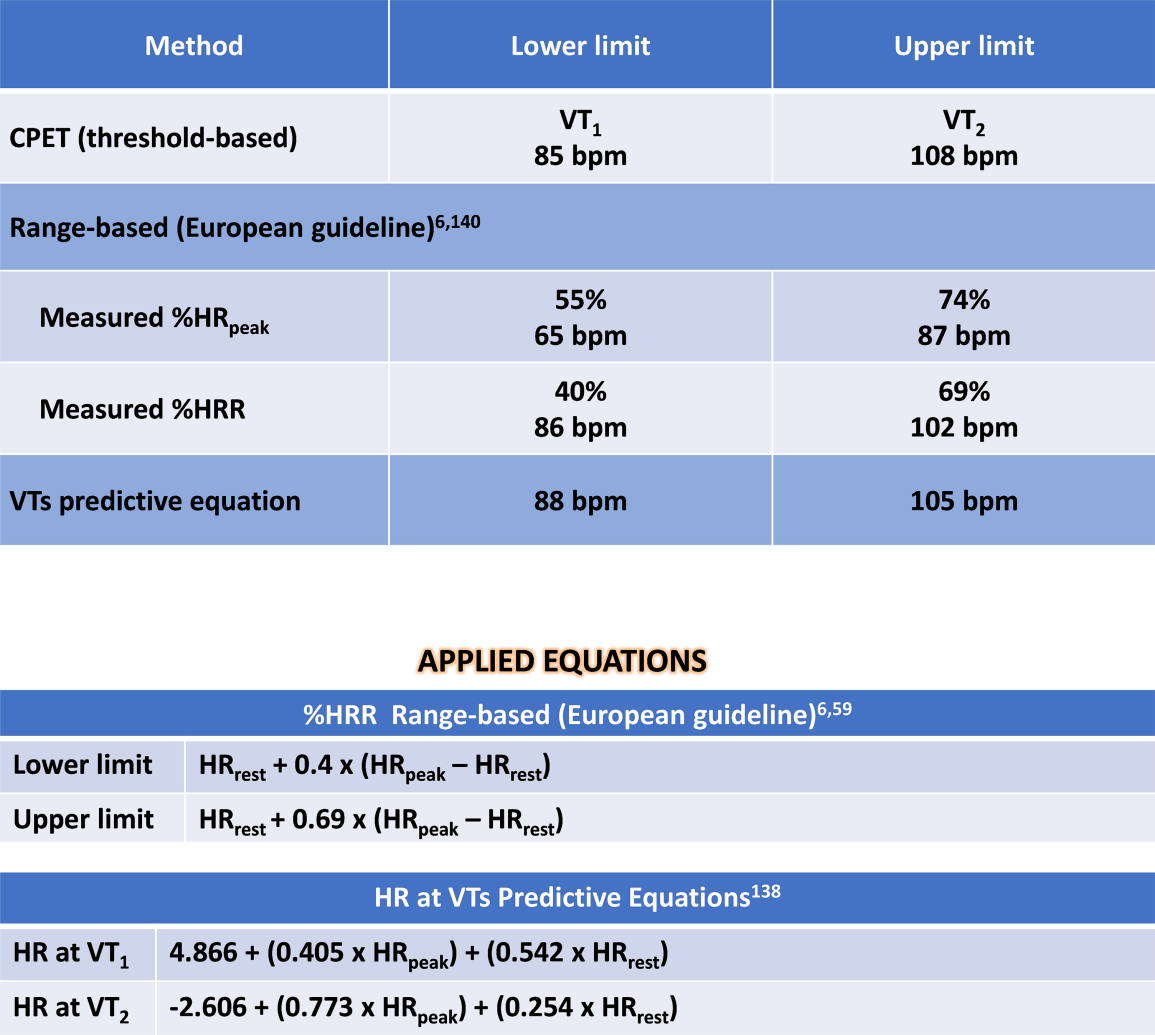
Solution of clinical case 2: prescribing moderate endurance exercise intensity using various heart rate-based approaches. CPET, cardiopulmonary exercise test; HR, heart rate; HR_peak_, peak heart rate; HRR, heart rate reserve; HR_rest_, rest heart rate; VO_2_, oxygen uptake; VTs, ventilatory thresholds; VT_1_, first ventilatory threshold; VT_2_, second ventilatory threshold; %HR_peak_, percentage of peak heart rate; %HRR, percentage oof heart rate reserve.

Figure 5 presents additional exercise test information, while Figure 6 illustrates the solution.

*Interpretation:*

The preferable method, guided by CPET's VTs, resulted in a moderate-intensity prescription ranging from 85 to 108 bpm (VT_1_ to VT_2_). Once again, the prescription based on %HR_peak_ exhibited lower accuracy, resulting in lower-intensity training recommendation compared to the threshold-based method. This discrepancy could lead to undertraining when relying on exercise prescription based on HR_peak_. Additionally, the prescription guided by both %HRR and equations demonstrated higher accuracy, despite presenting different numerical values, but closer to those identified by CPET.

## 8 Discussion

When collaborating with patients, healthcare professionals should integrate evidence, guidelines, theories, techniques, and tools related to behaviour change, as well as individual patients' requirements and values (57). It is crucial to select and implement the best possible approach, considering the available resources, with a continuous focus on strategies to enhance the quality of the delivery of CR, especially in low-resource settings.

It is advisable to implement a personalized approach that takes into account the patient's preferences and capabilities, allowing them to select their exercise intensity rather than imposing it. This becomes particularly vital when seeking sustained commitment over the long term (109).

Recognizing the importance of improving general physical activity advice, Festa et al. (113) recently suggested integrating the RPE scale, TT, and weekly training distribution as practical tools in the WHO guidelines for the general population to enhance the establishment of endurance training zones. An RPE < 11 (Borg 6–20) or <4 (Borg modified 0–10), or the ability to speak comfortably, signifies low-intensity exercise. Conversely, a Borg score ≥11 or Borg modified ≥4, or the inability to speak comfortably, indicates moderate to high-intensity exercise. Such approaches could provide valuable assistance to individuals in healthcare or training settings and professionals who lack adequate tools for creating exercise programs.

Therefore, optimizing the prescription of exercise intensity in CR settings and providing personalized physical activity advice, by correlating the intensity component with individual physiological changes promotes a more consistent and impactful exercise routine, effectively enhancing fitness levels among the general population and individuals with CVD (141). This additionally benefit decision support systems for CR, such as EXPERT tool, aiding in the integration of a defined methodology for assessing exercise intensity (142). Moreover, it contributes to enhancing access to healthcare services through the utilization of the internet and telemonitoring. This advancement is pivotal for the evolution of new practices in CR, including home-based training programs (143).

**Figure 7:**
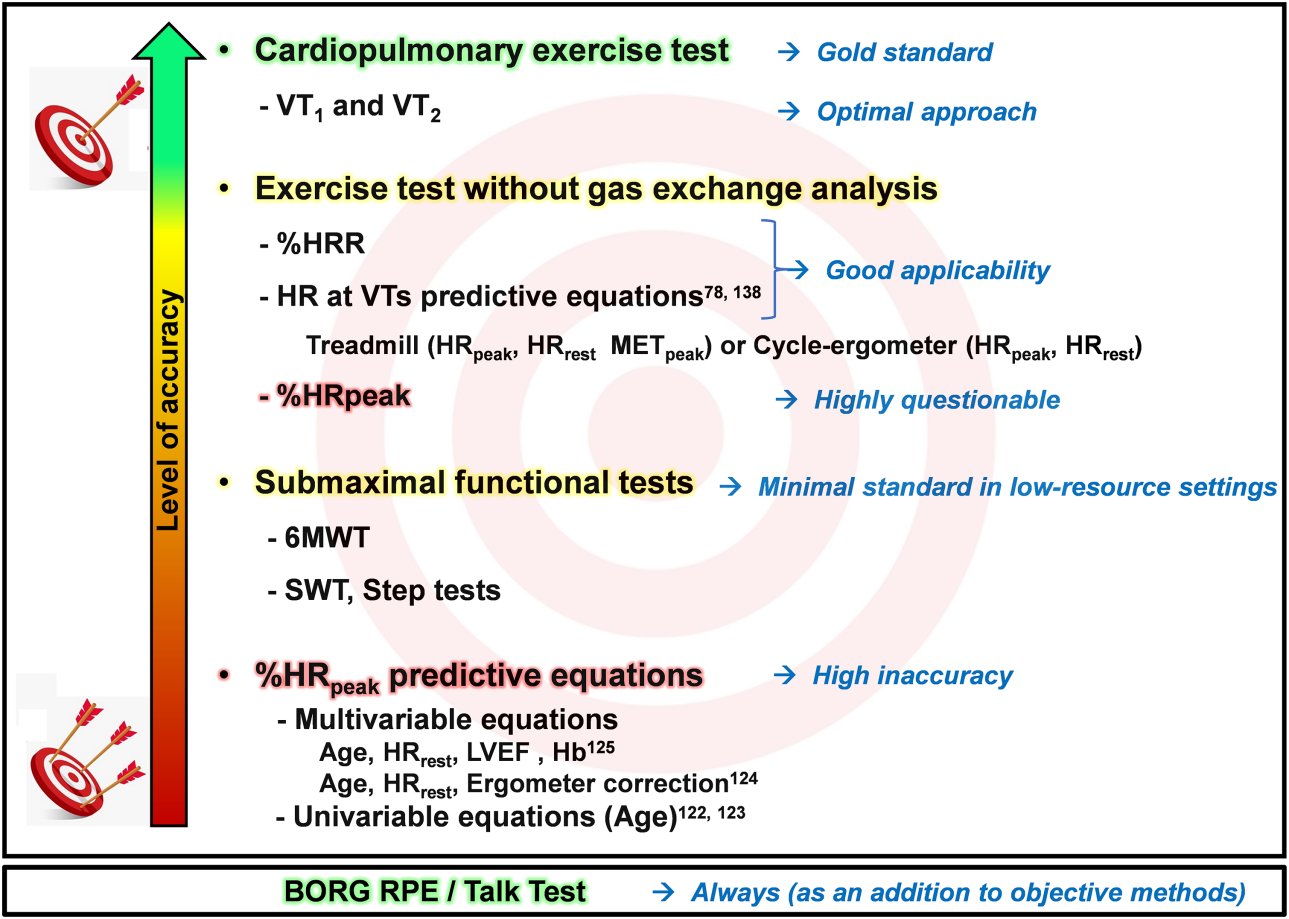
Schematic illustration of endurance exercise intensity methods, ranging from minimal to optimal standards, considering high and low resource settings. LVEF, left ventricular ejection; Hb, hemoglobin; HR, heart rate; HR_peak_, peak heart rate; HRR, heart rate reserve; HR_rest_, rest heart rate; MET_peak_, peak metabolic equivalent of tasks; SWT, shuttle walk test; VT_1_, first ventilatory threshold; VT_2_, second ventilatory threshold; %HR_peak_, percentage of peak heart rate; %HRR, percentage oof heart rate reserve; RPE, Ratings of Perceived Exertion; 6MWT, six-minute walk test.

Figure 7 illustrates, with increasing accuracy, the currently most recommended and commonly used methods for assessing and prescribing endurance exercise intensity in CR.

To sum up, this document has provided a practical and critical update on the prescription of endurance exercise intensity for CVD patients, encompassing worldwide guideline recommendations and addressing applicability in clinical practice across various resource availability realities, from minimal to optimal. The optimal approach is to guide intensity prescription based on VTs obtained via CPET. In the absence of CPET, the next best option is an individual assessment through an exercise test without gas exchange analyses. In low-resource settings where this option is also unavailable, performing at least another objective submaximal functional test, like 6MWT, can provide direct measure of the physical condition and should be the minimal approach. Additionally, objective methods should always be complemented by subjective ones, such as Borg RPE and TT, to adjust prescription intensity and consider patient preferences and motivations.
